# Unraveling the Causal Links Between Immune Traits and Hepatocellular Carcinoma: Insights From a Bi-Directional Mendelian Randomization Study

**DOI:** 10.5152/tjg.2025.24558

**Published:** 2025-04-21

**Authors:** Jie Li, Gechun Wang, Xiaonan Xiang, Jianguo Wang

**Affiliations:** 1 Department of Hepatobiliary Surgery, Affiliated Hangzhou First People’s Hospital, Westlake University School of Medicine, Hangzhou, China; 2 Department of Ophthalmology, Affiliated Xiaoshan Hospital, Hangzhou Normal University, Hangzhou, China; 3 Department of Colorectal Surgery, Sir Run Run Shaw Hospital, Zhejiang University School of Medicine, Hangzhou, China; 4 Department of Hepatobiliary Pancreatic Surgery and Minimally Invasive Surgery, Zhejiang Provincial People’s Hospital (Affiliated People’s Hospital), Hangzhou Medical College, Hangzhou, China

**Keywords:** Circulating immune traits, genetic associations, hepatocellular carcinoma, Mendelian randomization, prognosis

## Abstract

**Background/Aims:**

Hepatocellular carcinoma (HCC) is significantly influenced by the immune system, which plays a key role in its development, progression, treatment, and prognosis. While observational studies have revealed correlations between circulating immune traits and HCC, their genetic basis and causal links remain unclear. This study aims to investigate the genetic associations and bidirectional causal relationships between immune traits and HCC risk using Mendelian randomization (MR) approaches.

**Materials and Methods:**

Genome-wide association study summary statistics from the FinnGen cohort (R9, including 453 HCC cases and 287 137 controls) were used to perform a bidirectional two-sample MR analysis. The causal effects of immune traits on HCC, as well as reverse causality, were assessed. Sensitivity analyses, including heterogeneity and pleiotropy tests, were used to ensure the robustness and validity of the results.

**Results:**

Thirty-nine immune traits were identified to be significantly associated with HCC risk. Elevated levels of 10 immune traits were positively associated with increased HCC risk, while the abundance of 29 immune traits was inversely correlated with HCC incidence. Furthermore, the reverse MR analysis revealed significant causal effects of HCC on 11 immune traits.

**Conclusion:**

This study provides strong evidence of genetic links between systematic immune cell profiles and HCC, shedding light on the mechanisms underlying its onset and progression. These findings identify potential immune biomarkers for early diagnosis and immune-targeted therapies.

**Main Points**

- The study explored the causal relationship between immune cell profiles and hepatocellular carcinoma (HCC) using bidirectional Mendelian randomization, highlighting the genetic underpinnings of the immune system’s impact on HCC incidence and progression.
- The results demonstrated that elevated levels of 10 immune traits and reduced levels of 29 immune cell types are associated with HCC risk, while HCC also influences 11 specific immune traits.
- The study’s findings on immune cell profiles and HCC mechanisms could pave the way for improved early diagnosis and development of immune-based therapeutic strategies for HCC.

## Introduction

Hepatocellular carcinoma (HCC), the predominant primary liver malignancy, contributes significantly to the global burden of morbidity and mortality. The intricate etiology of HCC involves persistent liver inflammation driven by factors like viral hepatitis, excessive alcohol consumption, and non-alcoholic fatty liver disease.^1^ Evidence points to the critical role of chronic inflammation and immune dysfunction in HCC occurrence and progression.^2^

Within the HCC microenvironment, complex immune phenotypes emerge, featuring reduced (natural killer) NK cells and lymphocyte numbers with compromised function. Concurrently, suppressive immune cells such as regulatory T cells, myeloid-derived suppressive cells (MDSC), and tumor-associated macrophages increase, signaling immune evasion and treatment resistance.^3^ Additionally, dynamic changes in peripheral blood immune phenotypes mirror HCC progression and offer insights into patient prognosis. For instance, HCC patients with sustained hepatitis B virus (HBV) viral replication show decreased T cell frequency and elevated expression of inhibitory receptors, including T cell immunoreceptor with Ig and ITIM domains and T cell immunoglobulin domain and mucin domain-3.^4^ Notably, shifts in neutrophil and monocyte counts, along with decreased lymphocyte counts, correlate with disease stage progression and diminished survival.^5^

Recent large-scale population-based cohort studies indicated novel understanding of the intricate association between immune factors and HCC. Notably, studies propose a connection between autoimmune diseases (such as psoriasis, rheumatoid arthritis, and inflammatory bowel disease) and an elevated susceptibility to cirrhosis or HCC.^6^ Concurrently, apart from conventional serum markers like liver enzymes, contemporary investigations have underscored the role of several circulating inflammatory biomarkers in modulating liver cancer risk. For instance, positive correlations have been observed between C-reactive protein and interleukin-6 levels and the incidence of HCC.^7^ However, nuanced findings, as demonstrated by Yin et al’s^8^ work, introduce complexity to the relationship between circulating inflammatory factors and liver cancer development. Despite some controversy, these findings underscore the close association between chronic inflammation and circulating immune factors with liver tumorigenesis.

Mendelian randomization (MR), an analytical approach based on Mendelian inheritance, is a robust analytical approach widely employed for epidemiological causal inference. By using genetic variants as instrumental variables (IVs), MR helps to overcome common biases such as confounding and reverse causality, which often hinder observational studies. While MR analyses has been used to explore the relationships between inflammatory biomarkers and diverse diseases,^9^ its application in malignancies, particularly HCC, for assessing the role of circulating immune cell populations, remains relatively limited. Given the close link between chronic inflammation and HCC, it is crucial to systematically study the impact of immune cell heterogeneity on HCC development.

In this study, a bidirectional two-sample MR approach was employed to study the genetic association between circulating immune cell populations and HCC risk. This bi-directional design allows us to examine both the causal effect of immune traits on HCC and how HCC may, in turn, influence immune traits, providing a more comprehensive understanding of the relationships. This method is particularly valuable for addressing reverse causality, a common challenge in observational studies of chronic diseases like HCC.

The findings complement previous epidemiological studies and offer novel insights into the genetic mechanisms underlying HCC. By uncovering the causal relationships between immune traits and HCC, the findings may provide new therapeutic strategies and early diagnostic biomarkers for HCC.

## Materials and Methods

### Study Design

A bidirectional two-sample MR analysis was conducted to explore the potential causal effects of immune phenotypes and HCC. Mendelian randomization utilize genetic variations as IVs to represent exposures, allowing for causal inference by minimizing biases such as confounding and reverse causality. To ensure validity, IVs must satisfy 3 key assumptions: (1) The genetic variant must be strongly associated with the exposure (relevance assumption); (2) The genetic variant must not be associated with any confounders affecting both the exposure and the outcome (independence assumption); (3) The genetic variant must influence the outcome only through the exposure, with no alternative pathways (exclusion restriction assumption).

The analysis consisted of 2 main steps. First, a two-sample MR approach was used to assess the causal effects of immune phenotypes on HCC risk. Immune traits significantly associated with HCC risk were identified. Second, a reverse MR analysis was performed to evaluate whether the occurrence of HCC causally influenced immune traits. This bidirectional approach provides a comprehensive understanding of the relationship between immune phenotypes and HCC.

**Figure 1.**
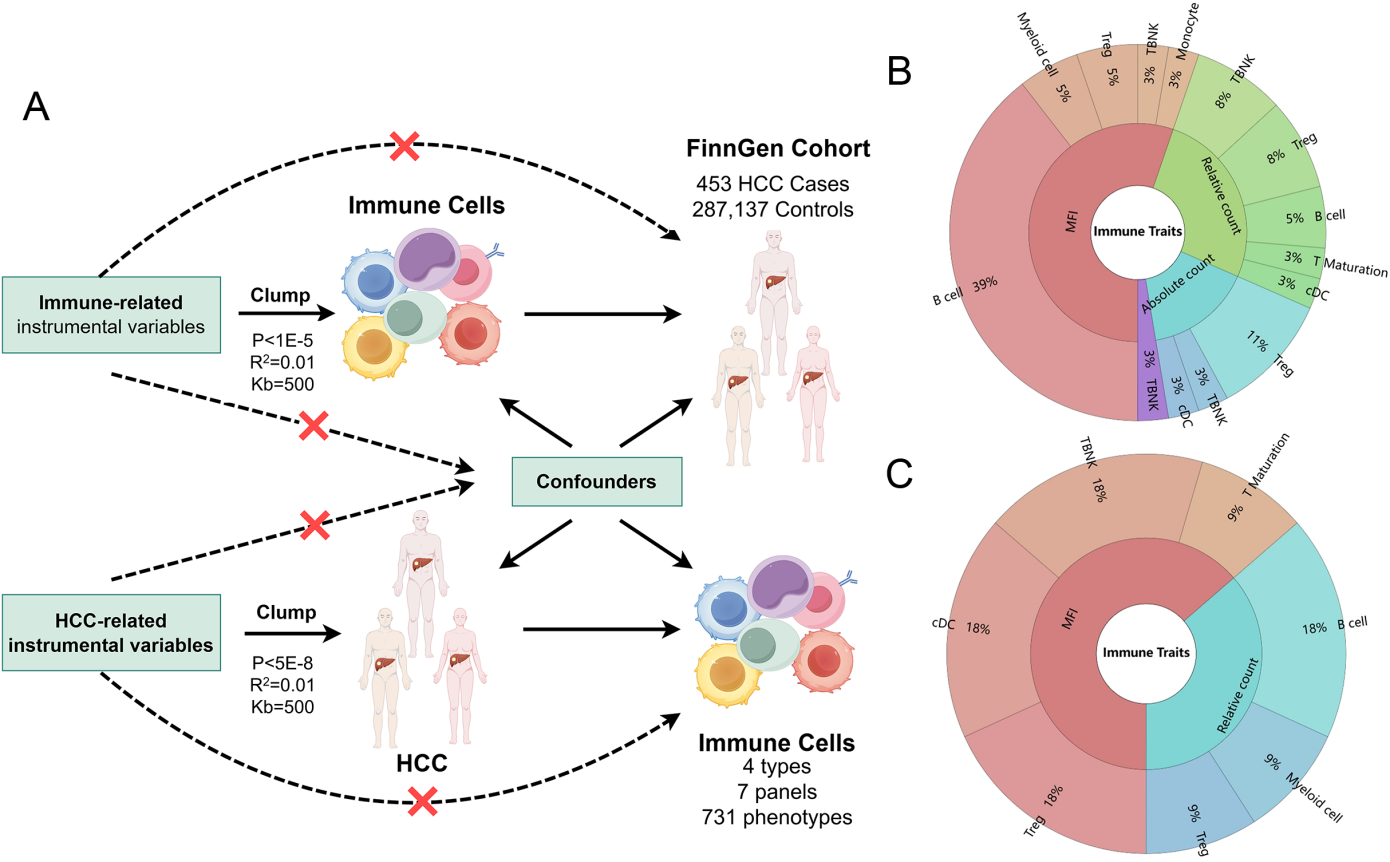
Study workflow and distribution of identified immune traits. A) A bidirectional two-sample MR analysis was conducted to investigate the causal relationships between circulating immune cell phenotypes and the risk of HCC. This analysis utilized data from the FinnGen R9 cohort, which included 453 HCC cases and 287 137 controls. B) Distribution of 39 immunophenotypes significantly associated with HCC pathogenesis. These include B cells (17 clusters), cDC (3 clusters), T cell maturation stages (1 cluster), monocytes (1 cluster), myeloid cells (2 clusters), TBNK (6 clusters), and Treg panels (9 clusters). C) Distribution of 11 immunophenotypes significantly affected by HCC. These consist of B cells (2 clusters), cDC (2 clusters), T cell maturation stages (1 cluster), myeloid cells (1 cluster), TBNK (2 clusters), and Treg panels (3 clusters).

With single-nucleotide polymorphisms (SNPs) used as IVs for the putative risk factor, MR analyses employ Mendel’s second law of independent genetic inheritance of alleles as a fundamental principle analogous to the random medication treatment in an randomized controlled trial. The study’s design and progression are illustrated in Figure 1A.

### Genome-Wide Association Study Data Sources

In this study, summary-level data from publicly available genome-wide association study (GWAS) datasets across multiple cohorts and consortia were utilized to investigate the causal relationships between immune phenotypes and HCC.

For the analysis of immune phenotypes, GWAS summary data from Orrù et al^10^ were accessed, which included 731 immunotypes derived from 3757 European individuals. This dataset provides a comprehensive set of immune traits, categorized into 118 absolute cell counts (AC), 389 median fluorescence intensities, 32 morphological parameters, and 192 relative cell counts. For HCC data in European populations, we obtained summary-level genetic data from the FinnGen R9 database (https://r9.finngen.fi/), comprising 453 HCC cases and 287 137 controls of European ancestry.

To complement the European data, GWAS summary statistics from the Biobank Japan (BBJ) cohort (http://jenger.riken.jp/en/) were utilized to analyze immune phenotypes and HCC in East Asian populations. The BBJ datasets include immune traits such as basophil count, eosinophil count, lymphocyte count, monocyte count, neutrophil count, and white blood cell (WBC) count derived from 62 076 individuals of Japanese ancestry. For HCC, the BBJ cohort provided genetic data for 1868 cases and 195 745 controls.

All relevant GWAS data for HCC are summarized in Table 1. All patient’s data were obtained from the public database. This analysis of publicly available data does not require informed consent and ethical approval.

### Selection of Instrumental Variables

To identify valid SNPs as IVs for immune cell traits, a significance threshold of *P* < 1 × 10^−^^5^ was applied. For SNPs associated with HCC, a more stringent threshold of *P* < 5 × 10^−8^ was used.

To minimize bias from linkage disequilibrium, clumping was performed using PLINK, with a threshold of *R*^2^ < 0.01 and a distance of 500 kb using the 1000 Genomes Project as a reference panel.^11^ The strength of the IVs was assessed using the F-statistics, calculated as: *F* = *R*^2^ (*n*-2)/1-*R* ^2^, where *R*^2^ represents the proportion of the exposure variance explained by the SNPs, and *n* is the effective sample size. Single-nucleotide polymorphisms with an F-statistic value below 10 were excluded to avoid weak instrumental strength. To further ensure validity, SNPs were screened using PhenoScanner v2 (http://www.phenoscanner.medschl.cam.ac.uk/).^12^ Single-nucleotide polymorphisms associated with potential confounders to HCC were excluded. The confounding factors included BMI, smoking, alcohol consumption, T2 diabetes, viral hepatitis, and chronic liver diseases such as NAFLD, cirrhosis and fatty liver.^13^

### Statistical Analysis

All analyses were conducted using R 4.3.1 software (R Foundation for Statistical Computing; Vienna, Austria). To explore causal relationships between immune phenotypes and HCC, established MR techniques were applied, including the inverse variance weighting (IVW) method, MR-Egger method, weighted median (WM) method and mode-based methods. These analyses were implemented using the R packages “TwoSampleMR” and “MendelianRandomization”.

To assess heterogeneity among the selected IVs, Cochran’s Q statistic was calculated. Horizontal pleiotropy was evaluated using the MR-Egger intercept, where statistical significance indicated the presence of pleiotropic effects.^14^ Additionally, the MR pleiotropy residual sum and outlier (MR-PRESSO) method was applied to detect and exclude outliers influenced by horizontal pleiotropy, which could otherwise distort causal estimates.

Positive MR results were defined by the following criteria: 1) Consistent beta direction across IVW, WM, MR-Egger, and MR-PRESSO analyses; 2) Statistically significant IVW test (*P* < .05). To further ensure the robustness of the findings, sensitivity analyses were performed, including scatter plots to visualize potential outliers, funnel plots to evaluate the absence of heterogeneity, and leave-one-out analyses to examine the influence of individual SNPs on the overall MR estimate.

## Results

### Causal Effect of Immunophenotypes on Hepatocellular Carcinoma

The analysis identified 39 immune traits with significant causal effects on HCC risk, using the IVW method. These 39 immune cells are categorized into 7 major groups: B cells (17 clusters), conventional dendritic cell (cDC) (3 clusters), T cell maturation stages (1 cluster), Monocytes (1 cluster), Myeloid cells (2 clusters), TBNK (6 clusters), and Treg panels (9 clusters) (Figure 1B).

**Figure 2.**
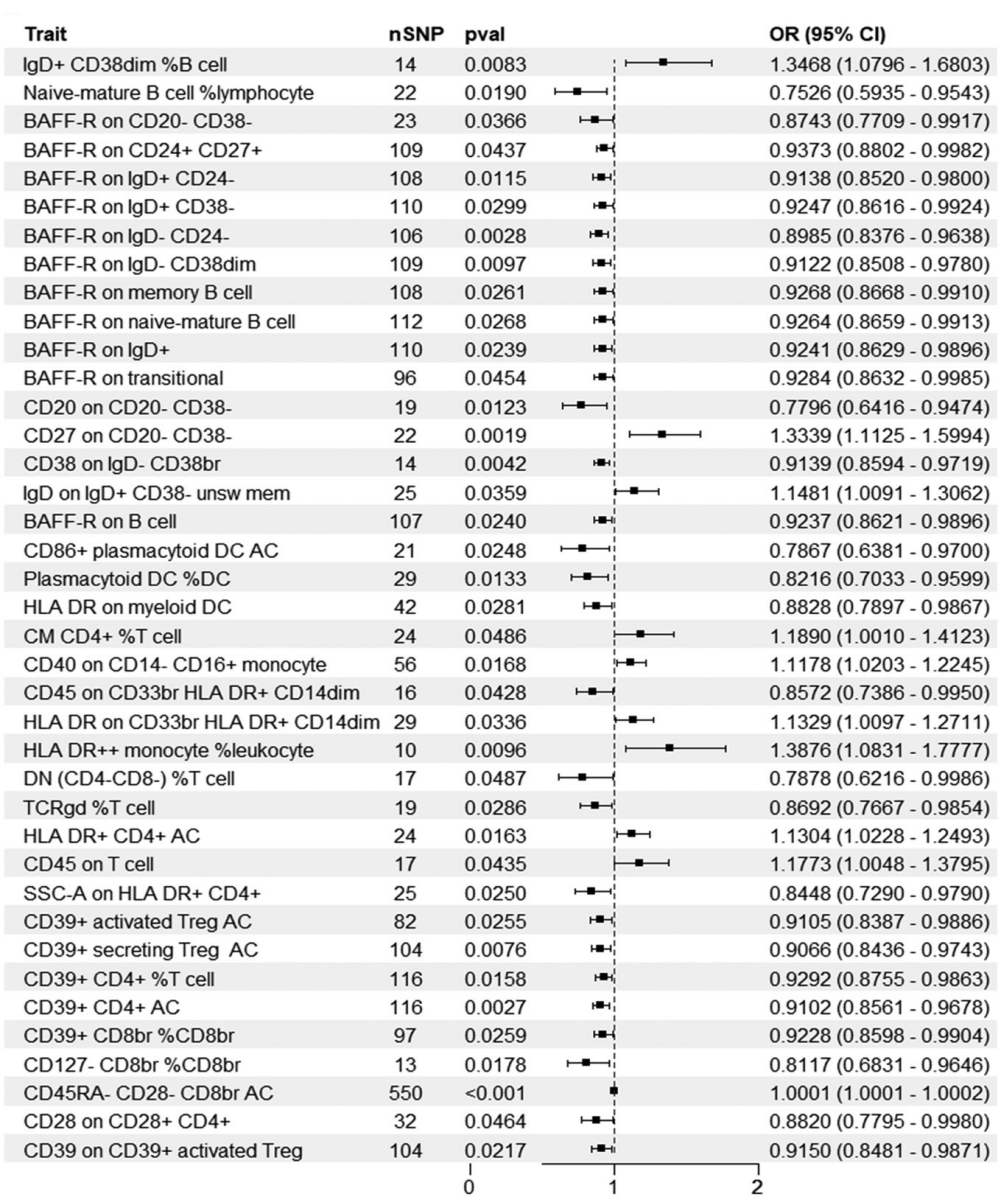
Causal effects of immune phenotypes on HCC. Thirty-nine immune phenotypes that exhibit significant causal effects on the occurrence of HCC were identified. Among these, 10 phenotypes were associated with promoting HCC, while 29 phenotypes exhibited protective effects by inhibiting HCC development. The forest plot provides detailed information for each immune phenotype with a positive association, including the number of instrumental variables (nSNPs), odds ratio (OR), and *P*-value derived from the inverse variance weighting (IVW) method.

The forest plot in Figure 2 provides a visual representation of the impact of positive exposure on HCC incidence. Among the 39 traits, 10 were positively associated with increased HCC risk, suggesting a possible role in promoting tumor progression. Specifically, the observations revealed significant positive correlations between the incidence of HCC and the expression of IgD and CD27 on B cells, the expression of human leukocyte antigen-DR (HLA-DR) on myeloid cells and CD4+ T cells, the expression of CD40 on non-classical monocytes (CD14-CD16+), central memory (CM) CD4+ T cells, and the expression of CD45RA-CD28- on Treg cells. Conversely, 29 traits were negatively associated, indicating potential protective effects against HCC development. A reduction in the incidence of HCC was noted in relation to the expression of B-cell Activating factor of the TNF family Receptor (BAFF-R) on B cells, the expression of CD28 and CD39 on Treg cells, the number of CD127-negative Treg cells, the number of plasmacytoid dendritic cells (pDC), the number of naive-mature B cells, the double negative T (DNT) ratio, and the TCRgd T cell number. Additionally, associations between the risk of HCC and the expression of CD45 on T cells or myeloid cells, as well as the morphology of HLA+CD4+ T cells, were observed. (Supplementary Tables 1 and 2). Sensitivity analyses confirmed the robustness of these findings, showing no evidence of heterogeneity or pleiotropy (Supplementary Figure 1).

### Causal Effect of Hepatocellular Carcinoma Onset on Immunophenotypes

**Figure 3.**
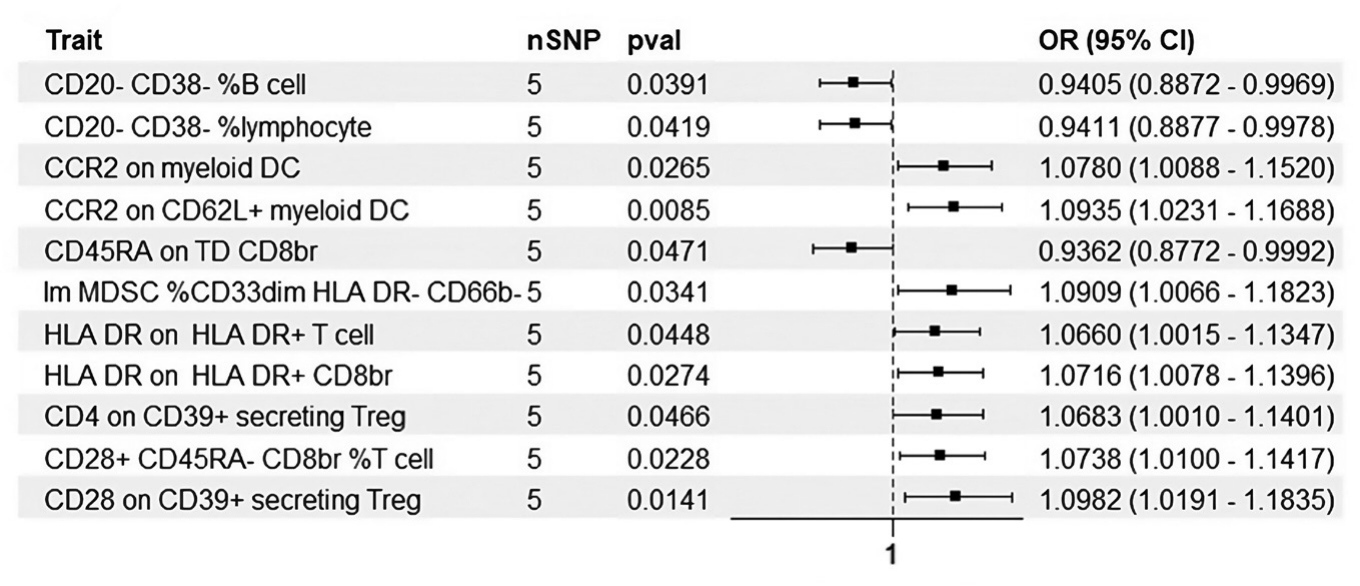
Causal effects of HCC on immune phenotypes. Eleven immune phenotypes were identified as being causally influenced by HCC. Among these, increased the levels of 8 immune traits and decreased the levels of 3 immune traits. The forest plot provides detailed information for each immune phenotype with a positive association, including the number of instrumental variables (nSNPs), odds ratio (OR), and *P*-value derived from the inverse variance weighting (IVW) method.

To examine whether HCC influences immune traits, a reverse MR analysis was conducted. Significant causal effects were observed for 11 immune traits, categorized into the following groups: B cells (2 clusters), cDC (2 clusters), T cell maturation stages (1 cluster), myeloid cells (1 cluster), TBNK (2 clusters), and Treg panels (3 clusters) (Figure 1C). As shown in Figure 3, the onset of HCC was associated with significant increases in the expression of CCR2 on cDCs, the expression of CD28 and CD4 on Tregs, and the expression of HLA-DR on T cells. Additionally, there was an elevation in the number of MDSCs. Conversely, the expression of CD20 and CD38 on B cells, and the expression of CD45RA on CD8+ T cells, exhibited reductions in the context of HCC development. Comprehensive analyses, including MR-Egger’s intercept and MR-PRESSO’s global test, effectively ruled out the presence of horizontal pleiotropy in these associations (Figure 3, Supplementary Tables 3 and 4). Sensitivity analyses supported the reliability of these results, with no evidence of heterogeneity or pleiotropy (Supplementary Figure 2).

### Bidirectional Causal Effect Between Immunophenotype and Hepatocellular Carcinoma in East Asian Populations

Using data from the BBJ cohort, the analysis was extended to investigate bidirectional relationships between immune phenotypes and HCC in East Asian populations.

**Figure 4.**
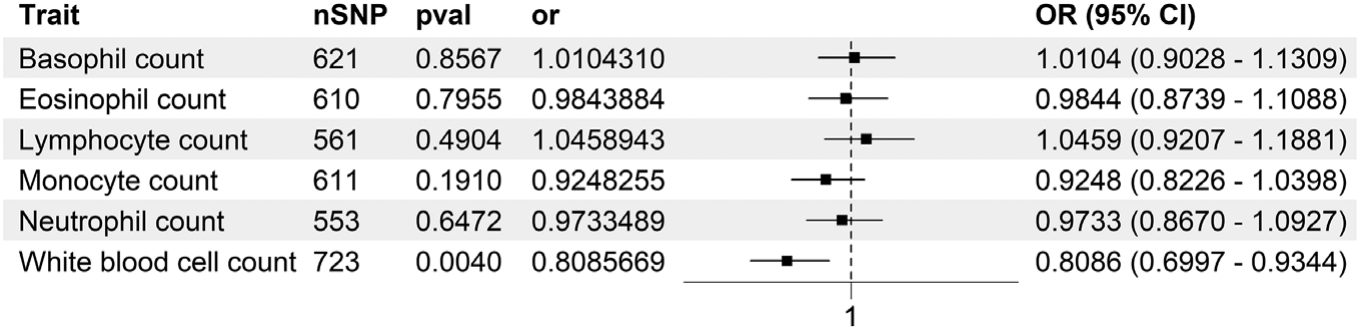
Causal effects among immune phenotypes and HCC in East Asian populations. The causal relationship between immune phenotypes, including eosinophils, basophils, monocytes, neutrophils, lymphocytes and WBCs, and HCC in an East Asian population (BBJ cohort) was explored. Among these, only WBCs showed a significant negative correlation with HCC. The forests plot provides detailed information for each immune phenotype, including the number of instrumental variables (nSNPs), odds ratio (OR), and *P*-value, as determined by the inverse variance weighting (IVW) method.

The results revealed a significant causal association between WBCs and the incidence of HCC. However, Cochran’s Q test indicated substantial heterogeneity in the WBC-HCC relationship, suggesting variability in the genetic associations. No significant causal links were identified for other immune components, including eosinophils, basophils, neutrophils, lymphocytes, and monocytes (Figure 4, Supplementary Table 5).

In the reverse analysis, no significant causal relationship was observed between HCC and immune traits, and no SNPs met the threshold for statistical significance.

## Discussion

Hepatocellular carcinoma, a frequently diagnosed and life-threatening cancer, presents challenges due to late-stage diagnosis, resulting in limited therapeutic options and unfavorable survival outcomes. Recent investigations underscore the significance of systemic immune alterations in tumor development, highlighting their potential as biomarkers for diagnosis and targets for treatment. In this study, MR was employed to explore the genetic foundations of immune cell involvement in the HCC development. The results identified 39 immune cell types significantly associated with HCC risk and 11 immune cell types implicated in its progression. These findings provide valuable genetic insights into the role of immune cells in HCC and may contribute to advancing diagnostic approaches and therapeutic strategies. In Table 2, the significant changes revealed for each immune cell in HCC are summarized (Table 2).

The proper development and activation of B cells play a pivotal role in maintaining homeostasis and facilitating effective responses against pathogens.^15^ The study reveals distinct associations between the functional states of B cells and the risk of HCC. Notably, the expression of IgD and CD27 on B cells, markers associated with memory B cells, was linked to an increased HCC incidence. Conversely, the presence of BAFF-R, a key receptor crucial for B cell survival and maturation, and the existence of naive-mature B cells were associated with a reduced HCC risk. These findings align with recent studies. Dong et al^16^ identified CD27 as a soluble immune checkpoint, showing a positive correlation with the incidence of HCC in patients with sustained viral response to HCV. Similarly, reduced BAFF-R expression on B cells has been observed in the peripheral blood of patients with HBV-associated HCC, implicating impaired B cell maturation as a critical factor in HCC progression.^17^

Myeloid-derived suppressive cells represents a diverse population of immature and immune-suppressive myeloid cells. These cells are recruited by tumors and infiltrate the tumor microenvironment, where they play a critical role in promoting immune evasion.^18^ Recent studies have reported increased MDSC levels in the peripheral blood of individuals with HCC compared to healthy individuals and those with chronic liver disease. This accumulation is often associated with poor clinical outcomes.^19^ The study highlights the involvement of myeloid cell phenotypes in peripheral blood during the onset of HCC. Elevated levels of HLA-DR+ on monocytes were associated with an increased incidence of HCC, consistent with findings in advanced HCC patients showing increased CD33+ and HLA-DR+ myeloid cells.^20^ Furthermore, non-classical monocytes expressing HLA-DR and CD40, characterized by CD14dim and CD16+, were linked to increased incidence of HCC, suggesting a role in enhanced activation and antigen presentation. In contrast, a significant negative association was observed between HCC incidence and the number of CD86+ pDCs. This finding suggests that reduced pDC levels may contribute to a weakened immune response, thereby increasing HCC risk.^21^

The study has uncovered significant associations between T cell characteristics and the risk of HCC. Among these, Treg phenotypes demonstrated notable links to HCC, CD127-, CD28, and CD39 Tregs were associated with protective effects, while CD45RA-CD28- Tregs were linked to an increased risk of HCC. The protective role of CD28, which facilitates the differentiation of Tregs from naive CD4 T cells, and the ATP-hydrolyzing function of CD39, indicative of highly active suppressive Tregs, highlight their critical role in mitigating the progression from inflammation to cancer.^22^ These findings suggest that functional Tregs may help maintain immune homeostasis, reducing the risk of HCC.^23^ Conversely, Treg dysfunction may elevate the risk of HCC incidence.

Moreover, T cell CM status was associated with an increased incidence of HCC, while higher numbers of γδ T cells and DNT cells were linked to a reduced risk of HCC, consistent with previous findings.^24^ The growing recognition of the crucial role of DNT/γδT cells in tumor immune surveillance has opened avenues for adoptive reinfusion therapies, including “off-the-shelf” options currently under investigation in clinical trials for various cancers, including HCC.^25^ These findings underscore potential therapeutic interventions in HCC based on T cell characteristics and immune surveillance mechanisms.

The progression of HCC induces notable changes in immune characteristics, particularly the upregulation of CD38 expression on B cells. As a multifunctional receptor and enzyme, CD38 plays a pivotal role in immune regulation by modulating signaling pathways that govern the activation, proliferation, and differentiation of B lymphocytes.^26^ While most studies on CD38 in HCC have focused on tumor-infiltrating immune cells, recent findings suggest a similar accumulation of CD38+ B cells in peripheral blood in other malignancies, such as breast cancer.^27^ These observations highlight the need for further investigation into the molecular mechanisms underlying CD38 expression on B cells and its potential clinical significance.

Simultaneously, this study reveals an association between the development of HCC and an increased presence of immature myeloid cells in peripheral blood, along with heightened expression of CCR2 on cDCs. The CCL2/CCR2 chemokine axis emerges as a critical pathway facilitating the recruitment of immature myeloid cells from peripheral blood to tumors. Once recruited, these myeloid cells are “domesticated” by tumors, contributing to immune evasion.^28^ Clinical studies have demonstrated the potential of therapeutic strategies targeting myeloid cell recruitment, which may inhibit tumor progression and enhance immunotherapeutic efficacy.^29^ The findings support the rationale for pursuing synergistic treatment strategies targeting this axis.

The development of HCC also significantly impacts on peripheral blood T cell phenotypes. A noteworthy increase in CD28+ and CD39+ regulatory T cells suggests an expanded pool of immunosuppressive Tregs. Elevated HLA-DR expression on T cells indicates T cell activation, albeit correlated with poorer postoperative survival rates.^30^ Intriguingly, the results uncover a dual role for activated Tregs in HCC pathogenesis. On one hand, a reduced Treg population intensifies the persistent inflammatory state, fostering HCC onset. On the other hand, HCC employs Treg-mediated immunosuppression to evade immune responses, aligning with its unique inflammatory-cancer transformation mechanism.^2^ Unraveling the intricacies of this finely tuned process will enhance our understanding of the pivotal role of Tregs in HCC’s development and potentially advance Treg-based immunotherapy.

In this study, two-sample Mendelian randomization analysis was employed, utilizing extensive GWAS cohorts with diverse racial backgrounds and a substantial sample size exceeding 300 000 individuals. This large-scale approach ensured robust statistical precision and enabled us to identify critical immune subgroups and potential immune targets implicated in HCC occurrence and progression. These findings provide valuable insights for future preclinical and clinical research.

Despite these promising results, certain limitations should be acknowledged. Firstly, the lack of individual-level data restricts the ability to perform stratified population analyses. Secondly, since the primary findings of the study relied on a European database (FinnGen), there may be limitations in generalizing the results to other ethnic groups. Although GWAS data from the BBJ cohorts were incorporated to investigate immune-HCC associations in East Asian populations, the imprecise classification of immune phenotypes in this dataset led to non-significant findings and heterogeneity issues.

Nevertheless, the clinical implications of this study remain noteworthy. While further validation is needed, the identified immune targets could contribute to the development of novel diagnostic markers and therapeutic strategies, offering a foundation for future research in HCC treatment.

To summarize, this study revealed the complex effect between peripheral immune landscape and HCC through bidirectional MR. As promising biomarkers and potential intervention targets, these peripheral immune subgroups are worthy of further clinical validation.

## Supplementary Materials

Supplementary Material

## Figures and Tables

**Table 1.** Study Population Characteristics and Sources

Phenotype	Ancestry	Sex	Participants Included	GWAS ID	nSNPs	Consortium	PMID
HCC	European	53 female	453 cases	C3_HEPATOCELLU_CARC_EXALLC	18 707 489	FinnGen R9	37840448
		400 male	287 137 controls				
HCC	East Asian	482 female	1866 cases	bbj-a-158	8 885 115	Biobank Japan	32514122
		1384 male	195 745 controls				
Imune phenotypes	European	Both sex	3580 individuals	From GCST90001391 to GCST90002121	Various	SardiNIA	32929287
Basophil count	East Asian	Both sex	62 076 individuals	bbj-a-12	6 108 953	Biobank Japan	29403010
Eosinophil count	East Asian	Both sex	62 076 individuals	bbj-a-20	6 108 953	Biobank Japan	29403010
Lymphocyte count	East Asian	Both sex	62 076 individuals	bbj-a-36	6 108 953	Biobank Japan	29403010
Monocyte count	East Asian	Both sex	62 076 individuals	bbj-a-41	6 108 953	Biobank Japan	29403010
Neutrophil count	East Asian	Both sex	62 076 individuals	bbj-a-44	6 108 953	Biobank Japan	29403010
White blood cell count	East Asian	Both sex	107 964 individuals	bbj-a-58	6 108 953	Biobank Japan	29403010

**Table 2.** Significant Changes Revealed for Each Immune Phenotypes in HCC

Exposure	Outcome	nSNP	b_IVW	*P*_IVW	OR (95% CI)
**1. Causal Effects of Immune Phenotypes on HCC (FinnGen R9)**					
IgD+ CD38dim B cell %B cell	Hepatocellular carcinoma (FinnGen R9)	14	0.297764	.008327951	1.35 (1.08-1.68)
Naive-mature B cell % lymphocyte		22	−0.28421	.018985877	0.75 (0.59-0.95)
CD86+ plasmacytoid dendritic cell absolute count		21	−0.23988	.024762144	0.79 (0.64-0.97)
Plasmacytoid dendritic cell %dendritic cell		29	−0.19645	.013320104	0.82 (0.70-0.96)
HLA DR++ monocyte %leukocyte		10	0.327582	.009550503	1.39 (1.08-1.78)
CD39+ activated CD4 regulatory T cell absolute count		82	−0.09372	.025491485	0.91 (0.84-0.99)
CD39+ secreting CD4 regulatory T cell absolute count		104	−0.09809	.007583867	0.91 (0.84-0.97)
Central memory CD4+ T cell %T cell		24	0.173121	.048621739	1.19 (1.00-1.41)
CD4-CD8- T cell %T cell		17	−0.23847	.048658755	0.79 (0.62-1.00)
TCRgd T cell %T cell		19	−0.14014	.02857448	0.87 (0.77-0.99)
HLA DR+ CD4+ T cell absolute count		24	0.12256	.016300065	1.13 (1.02-1.25)
CD39+ CD4+ T cell %T cell		116	−0.0734	.015765893	0.93 (0.88-0.99)
CD39+ CD4+ T cell absolute count		116	−0.09404	.002658014	0.91 (0.86-0.97)
CD39+ CD8+ T cell %CD8+ T cell		97	−0.08036	.025919334	0.92 (0.86-0.99)
CD127- CD8+ T cell %CD8+ T cell		13	−0.20861	.017791692	0.81 (0.68-0.96)
CD45RA- CD28- CD8+ T cell Absolute Count		550	0.000125	.000677519	1.00 (1.00-1.00)
BAFF-R on CD20- CD38- B cell		23	−0.13429	.036623807	0.87 (0.77-0.99)
BAFF-R on CD24+ CD27+ B cell		109	−0.06472	.043723185	0.94 (0.88-1.00)
BAFF-R on IgD+ CD24- B cell		108	−0.0902	.011539131	0.91 (0.85-0.98)
BAFF-R on IgD+ CD38- B cell		110	−0.07828	.029866201	0.92 (0.86-0.99)
BAFF-R on IgD- CD24- B cell		106	−0.10704	.002772804	0.90 (0.84-0.96)
BAFF-R on IgD- CD38dim B cell		109	−0.0919	.009726554	0.91 (0.85-0.98)
BAFF-R on memory B cell		108	−0.07598	.0261467	0.93 (0.87-0.99)
BAFF-R on naive-mature B cell		112	−0.0764	.026813186	0.93 (0.87-0.99)
BAFF-R on IgD+ B cell		110	−0.07892	.023947593	0.92 (0.86-0.99)
BAFF-R on transitional B cell		96	−0.0743	.045369937	0.93 (0.86-1.00)
CD20 on CD20- CD38- B cell		19	−0.24893	.012294858	0.78 (0.64-0.95)
CD27 on CD20- CD38- B cell		22	0.288113	.001862757	1.33 (1.11-1.60)
CD38 on IgD- CD38+ B cell		14	−0.09002	.004151047	0.91 (0.86-0.97)
IgD on IgD+ CD38- unswitched memory B cell		25	0.138117	.035888379	1.15 (1.01-1.31)
BAFF-R on B cell		107	−0.07942	.024024331	0.92 (0.86-0.99)
CD28 on CD28+ CD4+ T cell		32	−0.12555	.046437033	0.88 (0.78-1.00)
CD45 on T cell		17	0.16322	.04352297	1.18 (1.00-1.38)
CD40 on CD14- CD16+ monocyte		56	0.111326	.016758629	1.12 (1.02-1.22)
CD39 on CD39+ activated CD4 regulatory T cell		104	−0.08889	.021713432	0.91 (0.85-0.99)
CD45 on CD33+ HLA DR+ CD14dim		16	−0.15403	.04275627	0.86 (0.74-0.99)
SSC-A on HLA DR+ CD4+ T cell		25	−0.16863	.024959173	0.84 (0.73-0.98)
HLA DR on myeloid dendritic cell		42	−0.12471	.028137626	0.88 (0.79-0.99)
HLA DR on CD33+ HLA DR+ CD14dim		29	0.124766	.033604124	1.13 (1.01-1.27)
**2. Causal Effects of HCC (FinnGen R9) on Immune Phenotypes**					
Hepatocellular carcinoma (FinnGen R9)	CD20- CD38- %B cell	5	−0.0614	.039083502	0.94 (0.89-1.00)
	CD20- CD38- %lymphocyte	5	−0.0607	.04189785	0.94 (0.89-1.00)
	CCR2 on myeloid DC	5	0.07513	.026493492	1.08 (1.01-1.15)
	CCR2 on CD62L+ myeloid DC	5	0.08937	.00850036	1.09 (1.02-1.17)
	CD45RA on TD CD8br	5	−0.0659	.04709667	0.94 (0.88-1.00)
	Im MDSC %CD33dim HLA DR- CD66b-	5	0.087	.034056797	1.09 (1.01-1.18)
	HLA DR on HLA DR+ T cell	5	0.06391	.044772678	1.07 (1.00-1.13)
	HLA DR on HLA DR+ CD8br	5	0.06919	.027359944	1.07 (1.01-1.14)
	CD4 on CD39+ secreting Treg	5	0.06605	.046647831	1.07 (1.00-1.14)
	CD28+ CD45RA- CD8br %T cell	5	0.07124	.022801844	1.07 (1.01-1.14)
	CD28 on CD39+ secreting Treg	5	0.09369	.014076604	1.10 (1.02-1.18)
**3. Causal Effects of Immune Phenotypes on HCC in East Asian Populations (BBJ)**					
Basophil count	Hepatocellular carcinoma (BBJ)	621	0.010377	.856667312	1.01 (0.90-1.13)
Eosinophil count		610	−0.01573	.795528036	0.98 (0.87-1.11)
Lymphocyte count		561	0.044872	.490397747	1.05 (0.92-1.19)
Monocyte count		611	−0.07815	.191048818	0.92 (0.82-1.04)
Neutrophil count		553	−0.02701	.647171272	0.97 (0.87-1.09)
WBC count		723	−0.21249	.003978886	0.81 (0.70-0.93)

## Data Availability

The data involved in this article can be publicly available from the OpenGWAS (https://gwas.mrcieu.ac.uk/), GWAS Catalog (https://www.ebi.ac.uk/gwas/) and FinnGen (https://www.finngen.fi/fi). Code involved in manuscript can be accessed at https://github.com/xnxiang1995/MR.
