# Supplementary material for: Unraveling the Causal Links Between Immune Traits and Hepatocellular Carcinoma: Insights From a Bi-Directional Mendelian Randomization Study
Source: Turk J Gastroenterol. 2025 Apr 21;36(7):420–30. doi: 10.5152/tjg.2025.24558 (PMC12257684; doi:10.5152/tjg.2025.24558)
Supplement: Supplementary Material [file supplementary_material.pdf]

Supplementary Table 1. Summary of the results of Mendelian randomization analysis of the causal relationship between peripheral immune traits and hepatocellular carcinoma (FinnGen R9 cohort)

https://figshare.com/s/2d71c659624be677ecdb

Supplementary Table 2. Summary of the results of Mendelian randomization analysis of the causal relationship between identified 39 immune traits and hepatocellular carcinoma (FinnGen cohort)

https://figshare.com/s/2d71c659624be677ecdb

Supplementary Table 3. Summary of the results of Mendelian randomization analysis of the causal relationship between hepatocellular carcinoma and peripheral immune traits

https://figshare.com/s/2d71c659624be677ecdb

Supplementary Table 4. Summary of the results of Mendelian randomization analysis of the causal relationship between hepatocellular carcinoma and identified 11 immune traits

https://figshare.com/s/2d71c659624be677ecdb

Supplementary Table 5. Summary of the results of Mendelian randomization analysis of the causal relationship between immune traits and hepatocellular carcinoma (BBJ Cohort)

https://figshare.com/s/2d71c659624be677ecdb

Supplementary Table 6. Genotypic data of 39 identified immune traits that significantly influenced HCC occurrence

https://figshare.com/s/2d71c659624be677ecdb

Supplementary Table 7. Genotypic data of HCC that significantly influence immune traits

https://figshare.com/s/2d71c659624be677ecdb

GCST90001393

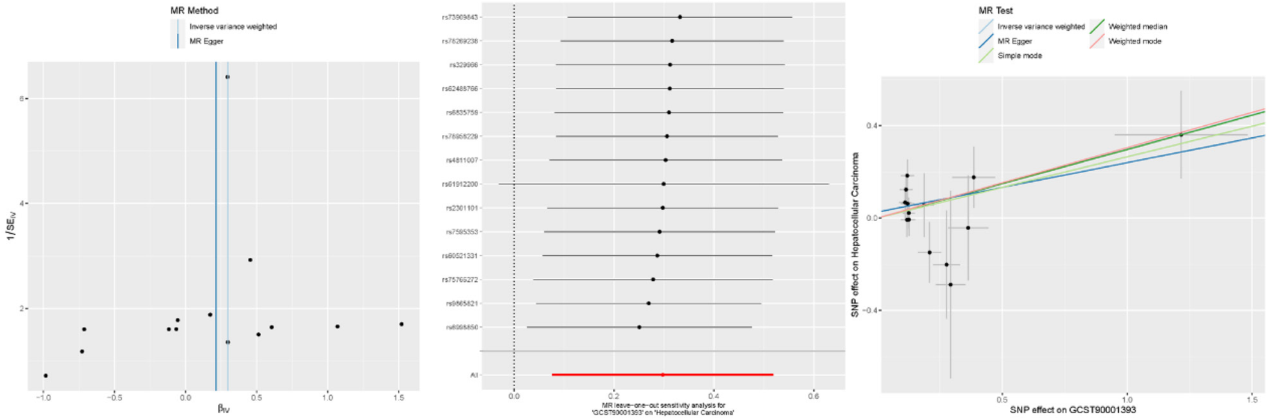

GCST90001437

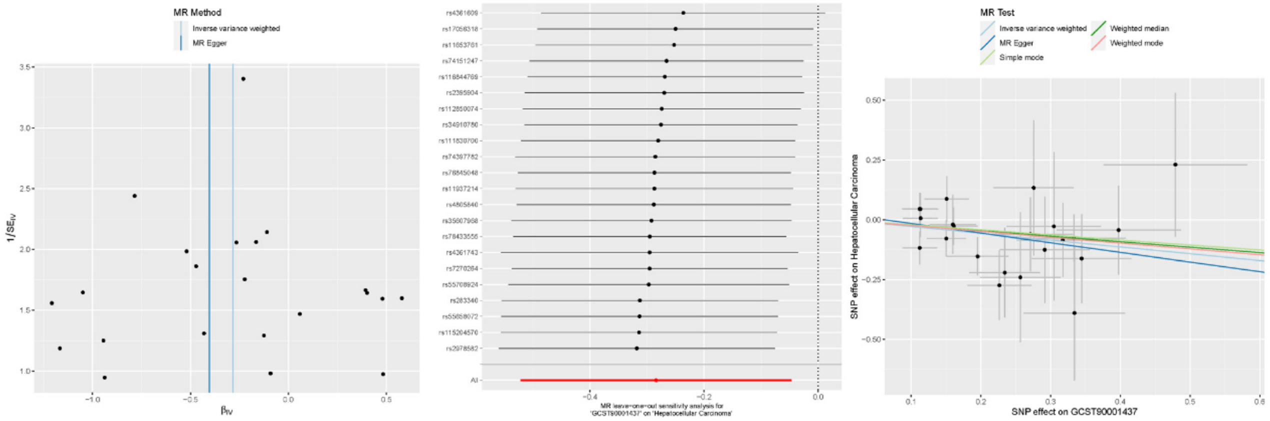

GCST90001466

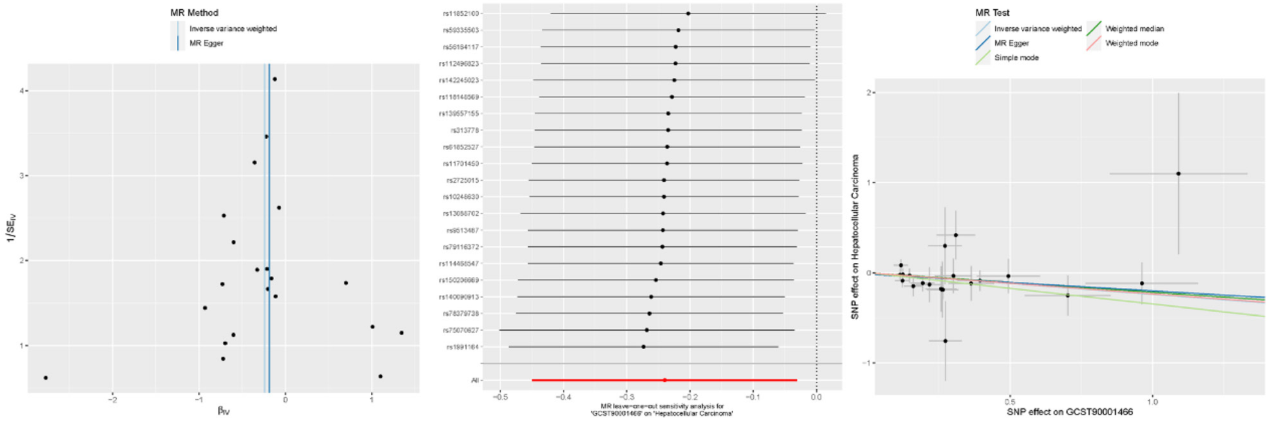

Supplementary Figure 1. Sensitivity Analyses for MR Results of Immune Traits and HCC. This figure presents the sensitivity analyses conducted to validate the MR results for the causal relationships between the 39 identified immune traits and HCC risk. Funnel plots, leave-one-out forest plots, and scatter plots were used to ensure the robustness of the MR estimates and identify potential biases.

GCST90001474

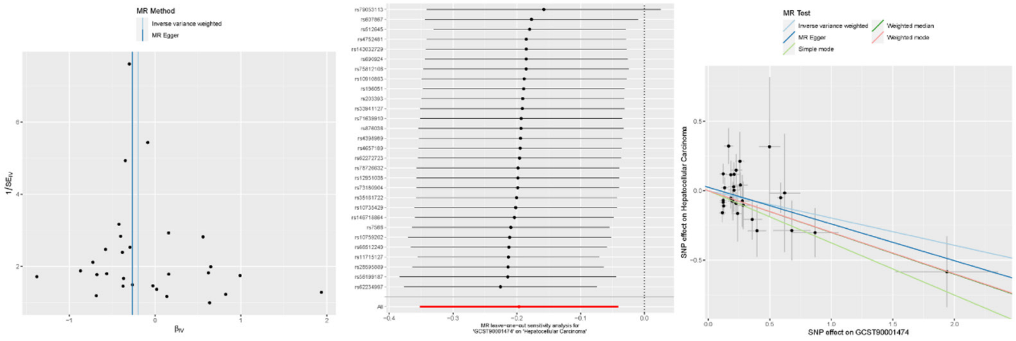

GCST90001476

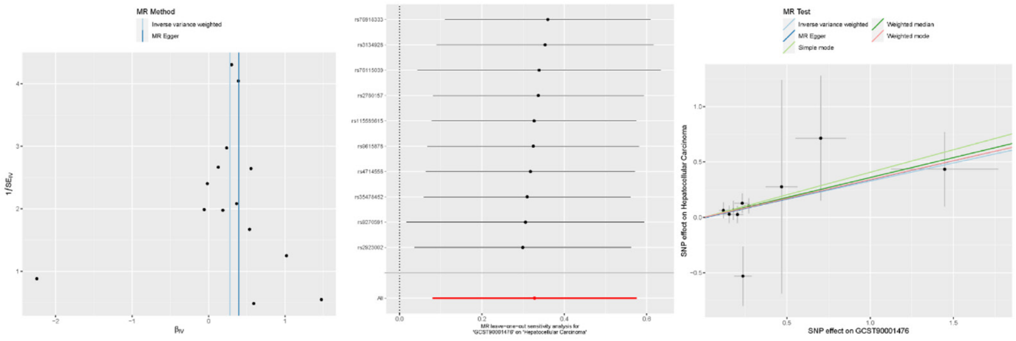

GCST9001489

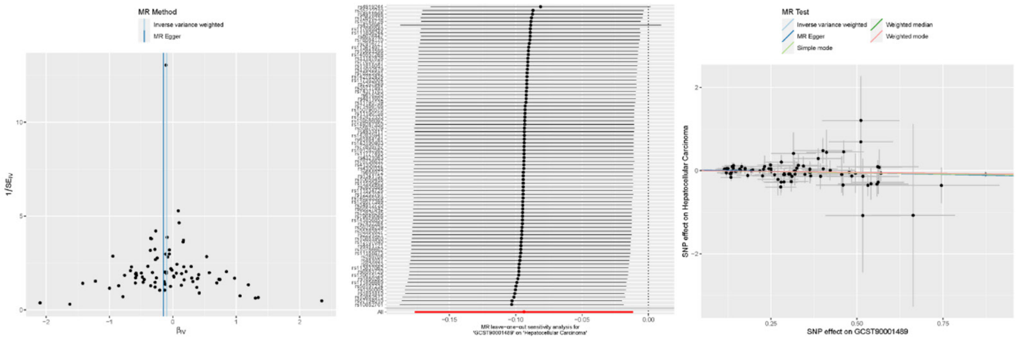

GCST90001495

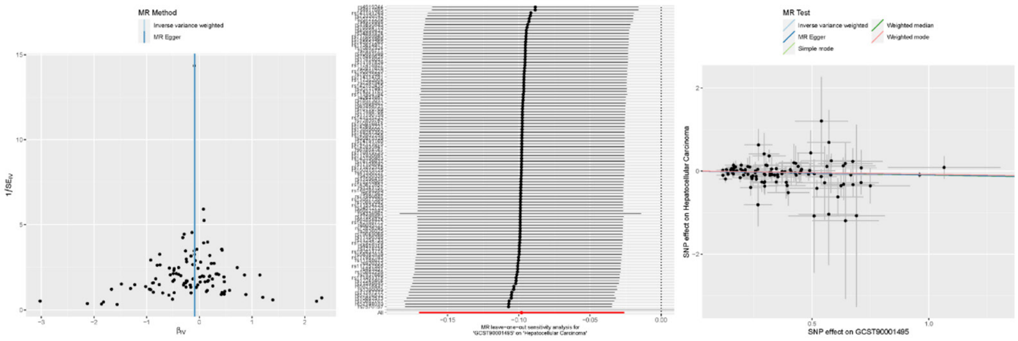

Supplementary Figure 1. (*Continued*).

GCST90001539

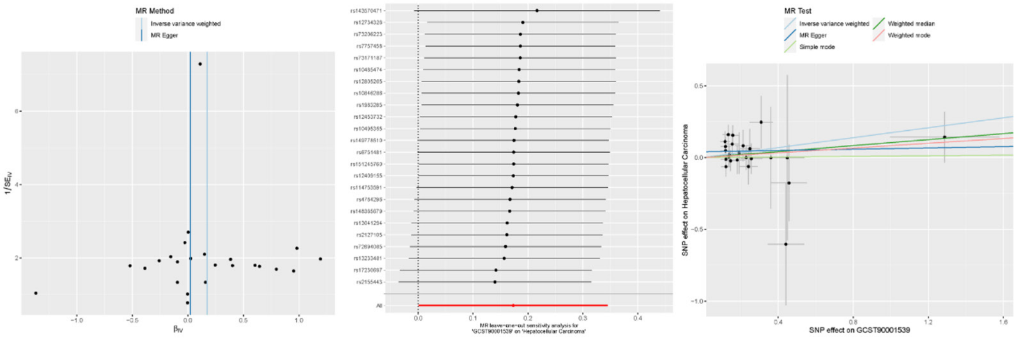

GCST9001599

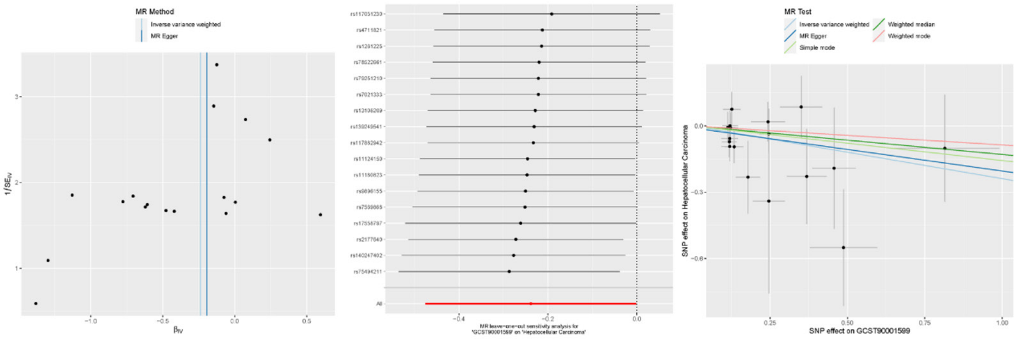

GCST90001616

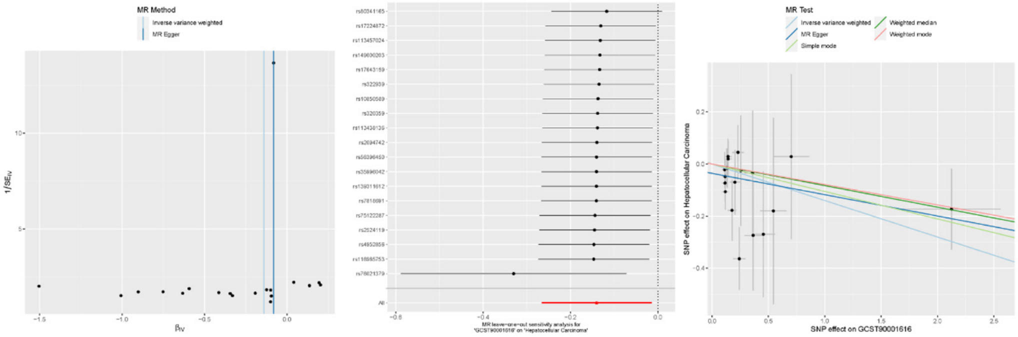

GCST90001624

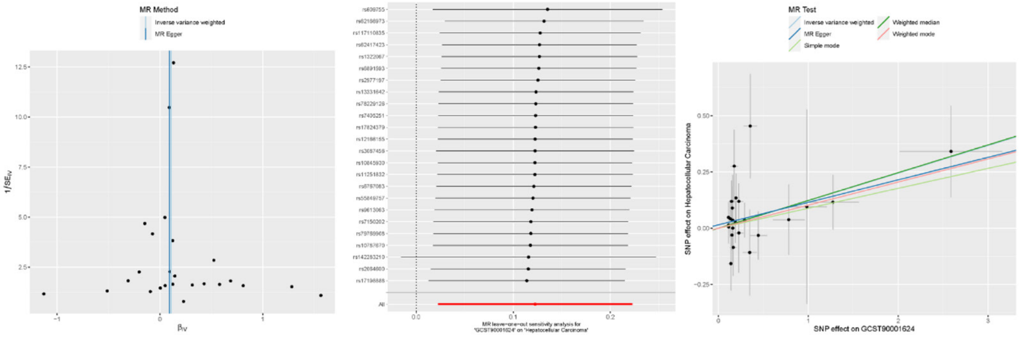

Supplementary Figure 1. (*Continued*).

GCST90001658

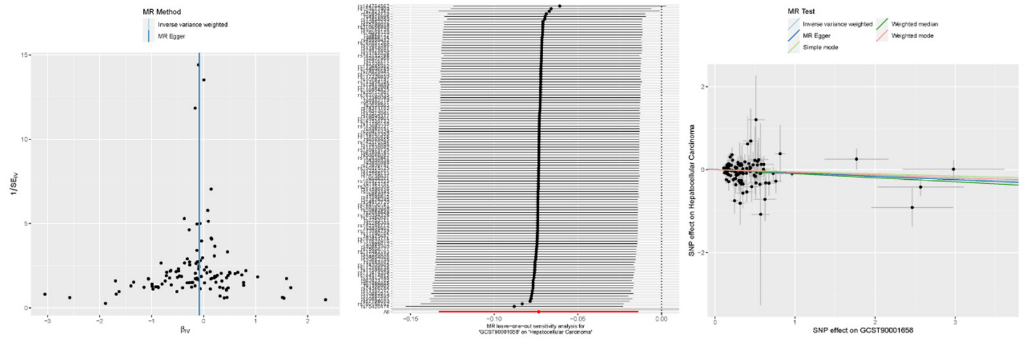

GCST90001660

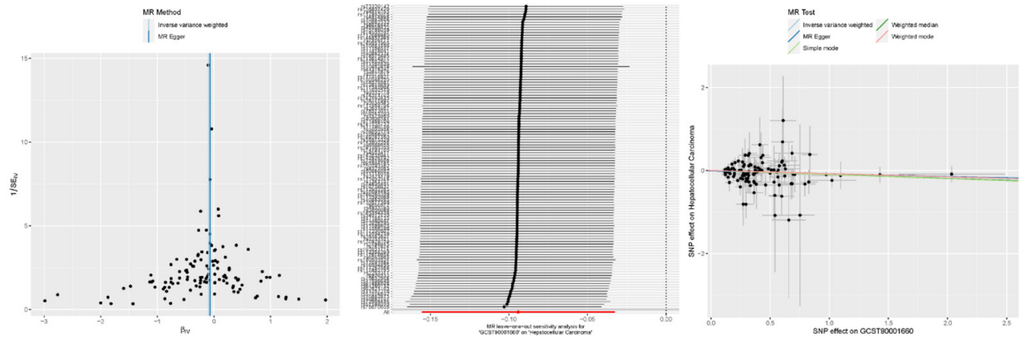

GCST90001671

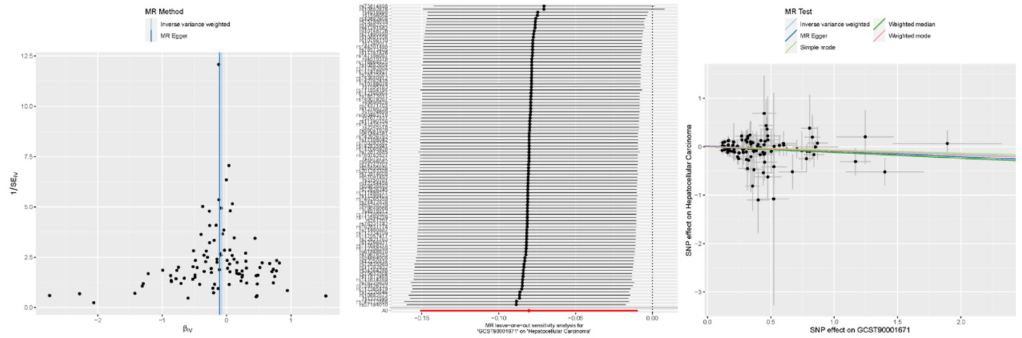

GCST90001683

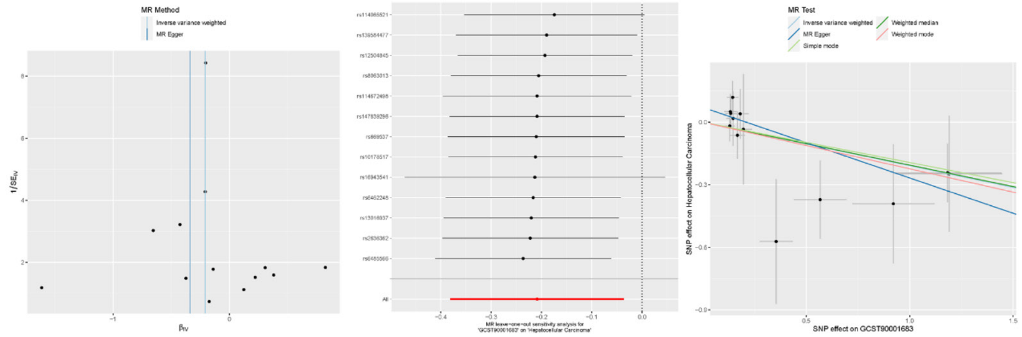

Supplementary Figure 1. (*Continued*).

GCST90001695

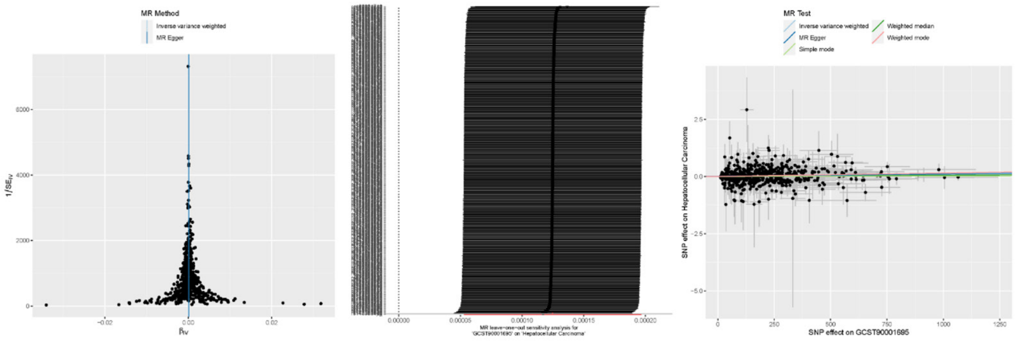

GCST90001701

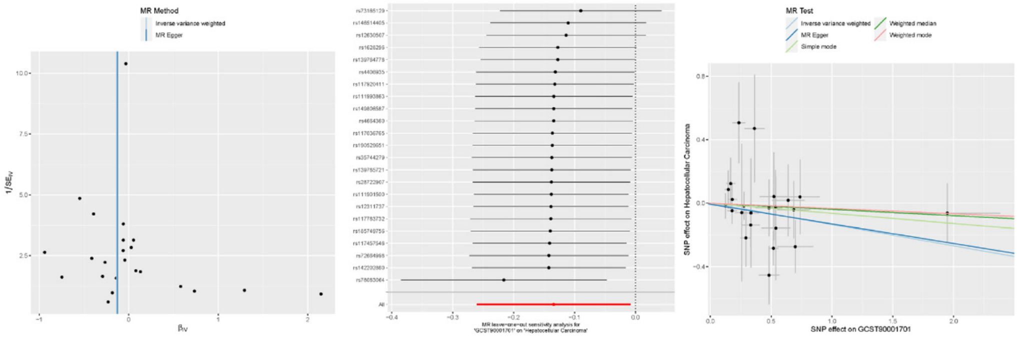

GCST90001702

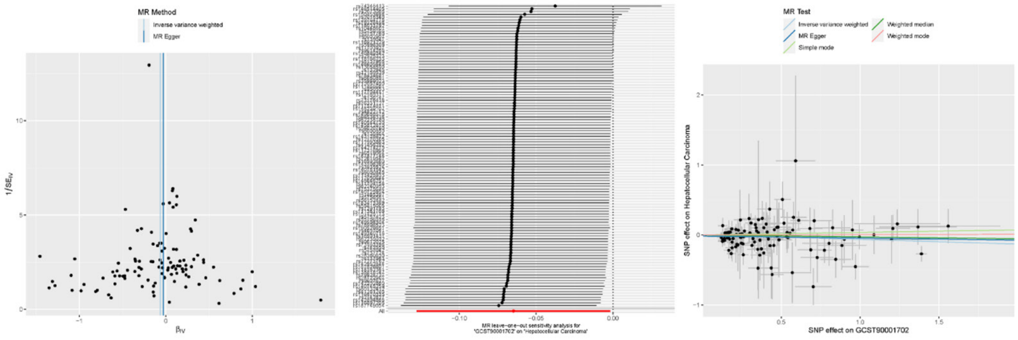

GCST90001704

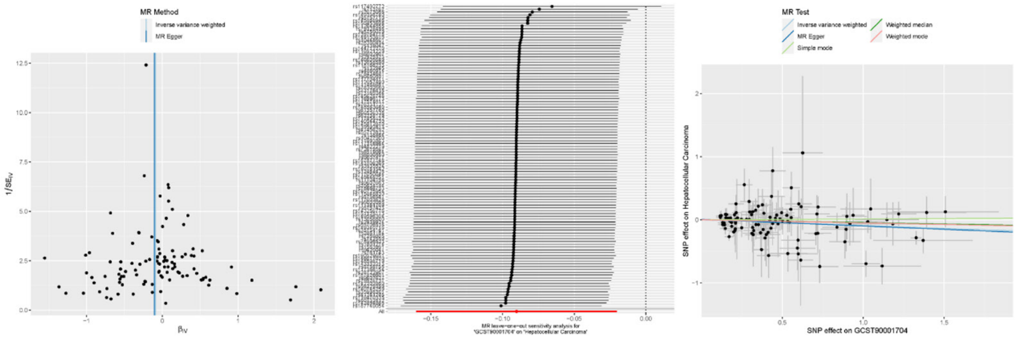

Supplementary Figure 1. (*Continued*).

GCST90001705

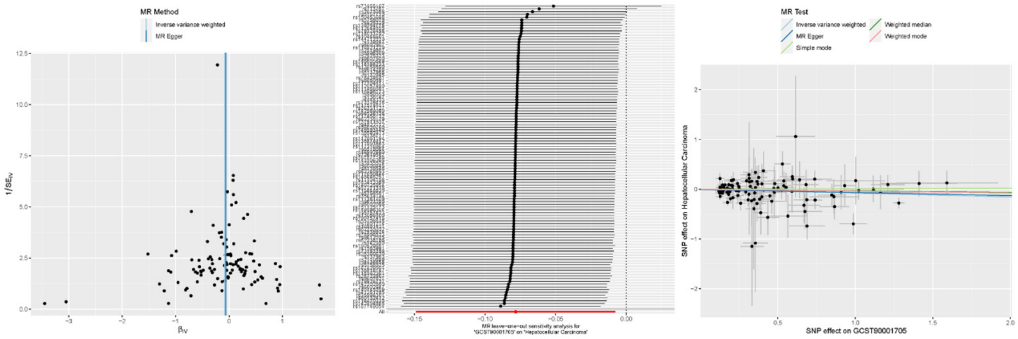

GCST90001710

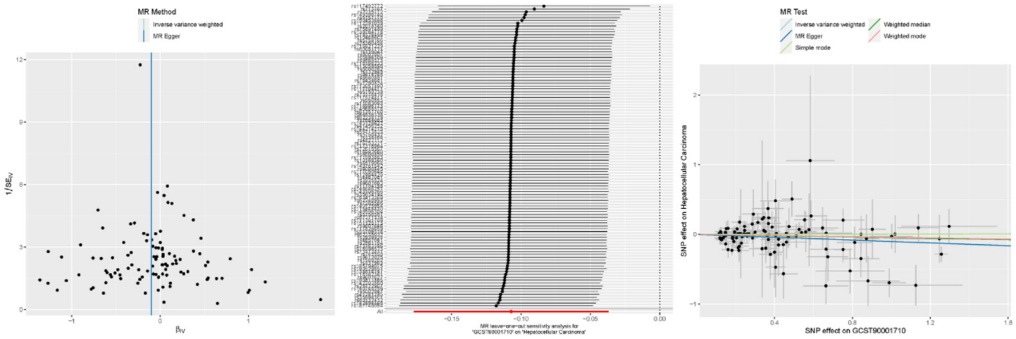

GCST90001714

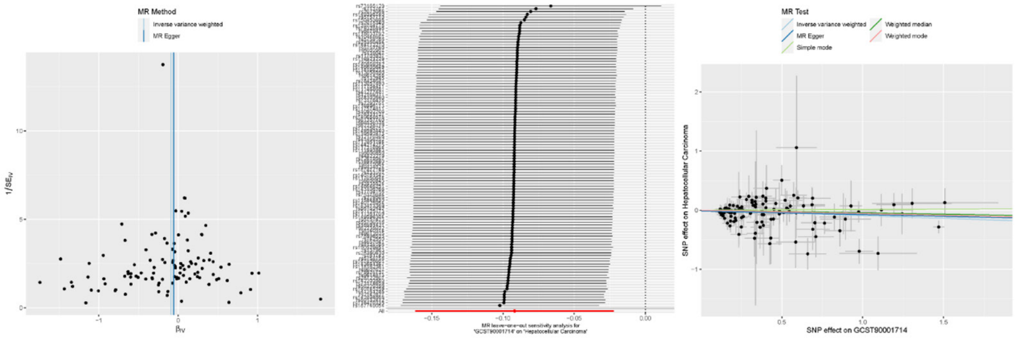

GCST90001715

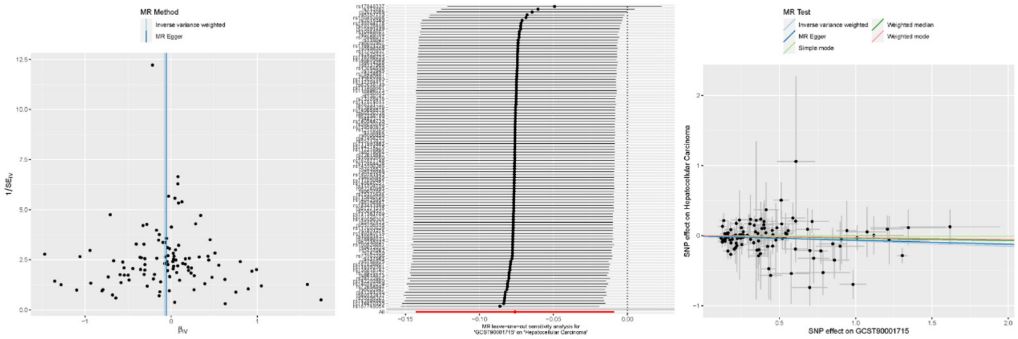

Supplementary Figure 1. (*Continued*).

GCST90001716

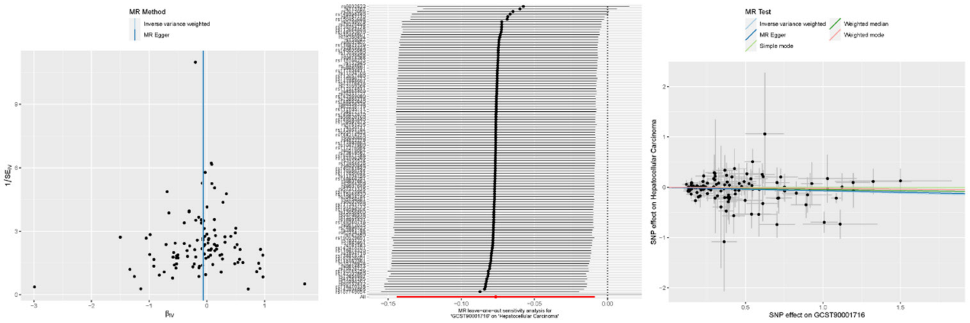

GCST90001719

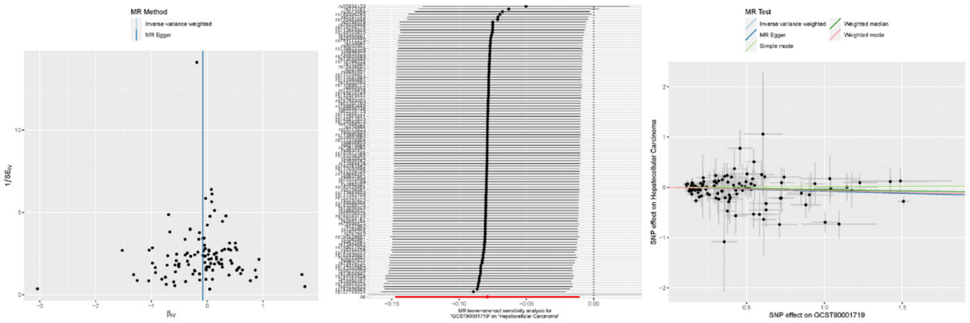

GCST90001720

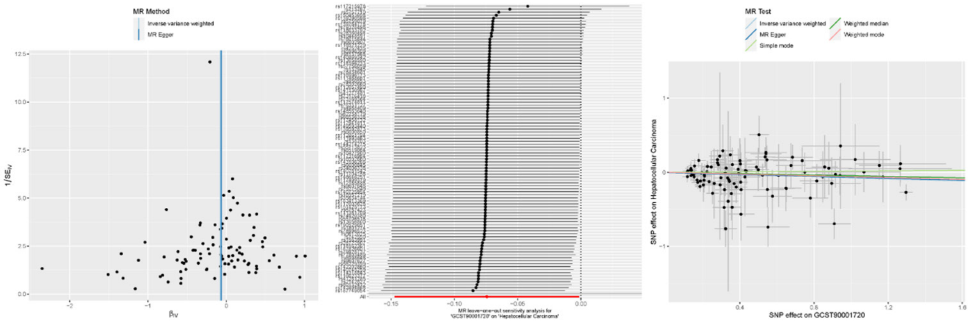

GCST90001744

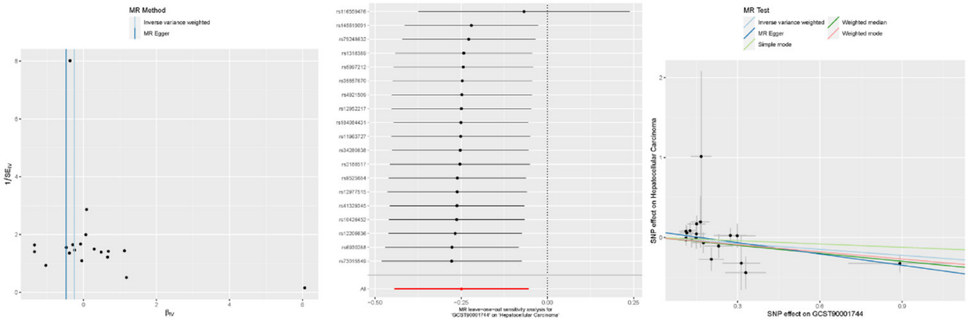

Supplementary Figure 1. (*Continued*).

GCST90001797

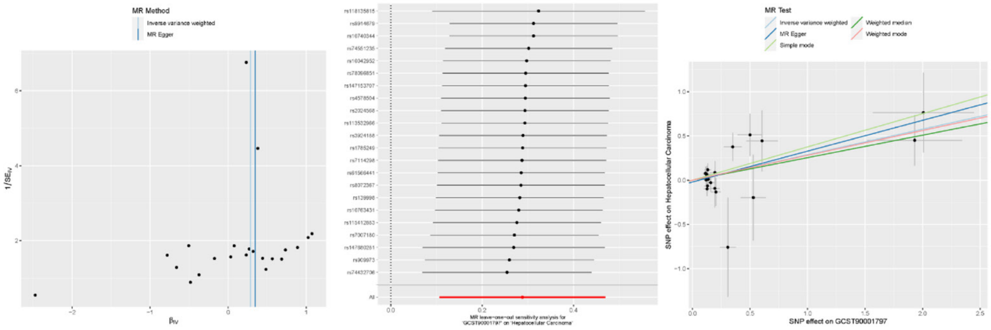

GCST90001814

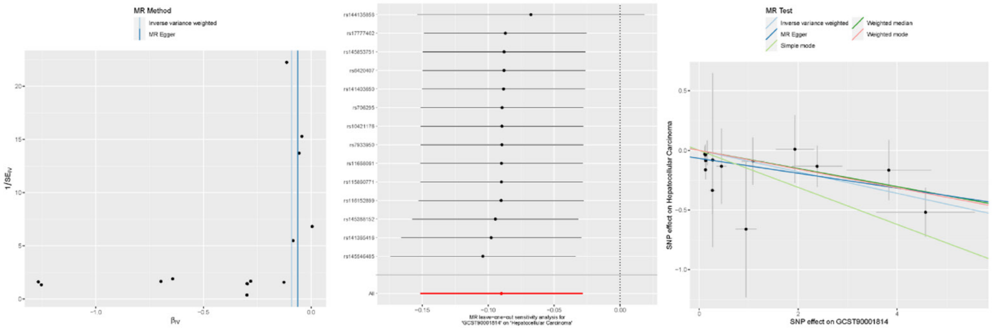

GCST90001823

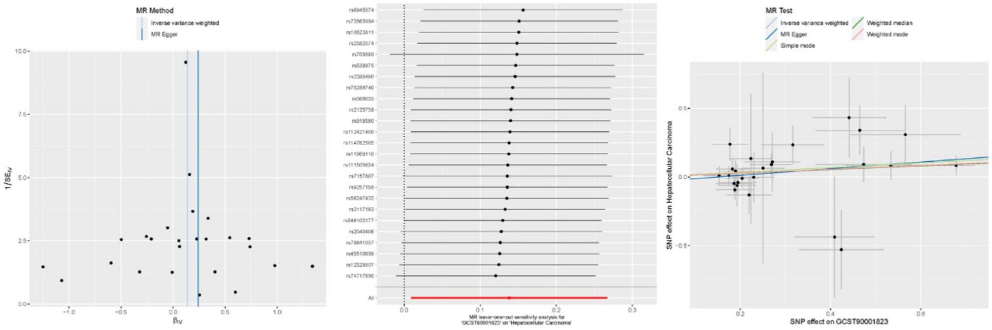

GCST90001829

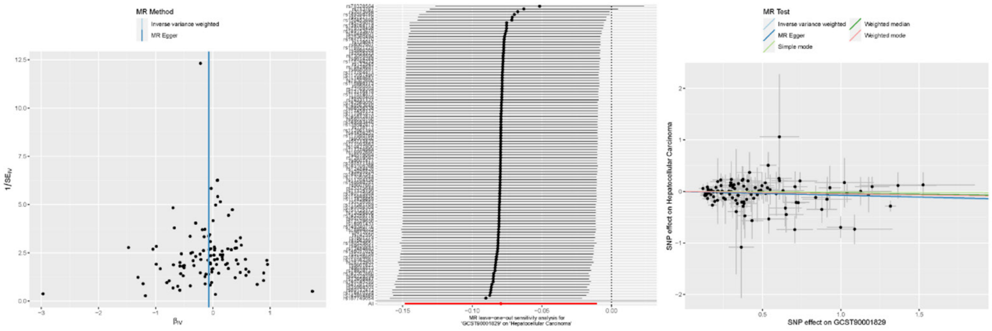

Supplementary Figure 1. (*Continued*).

GCST90001894

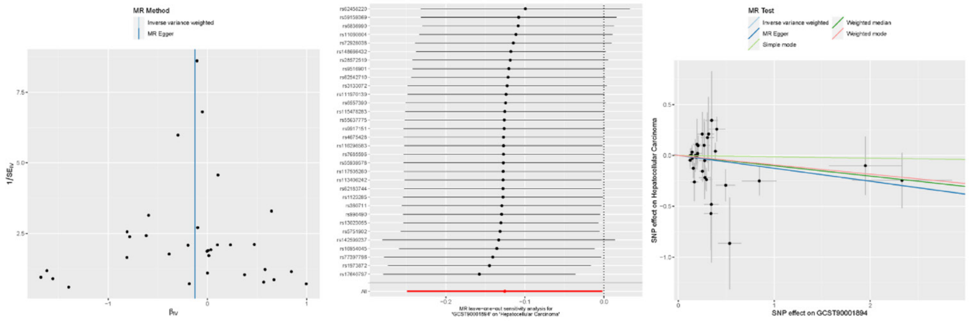

GCST90001915

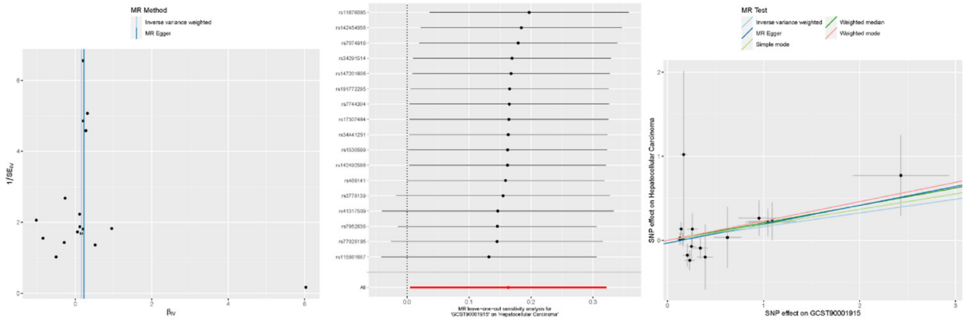

GCST90001989

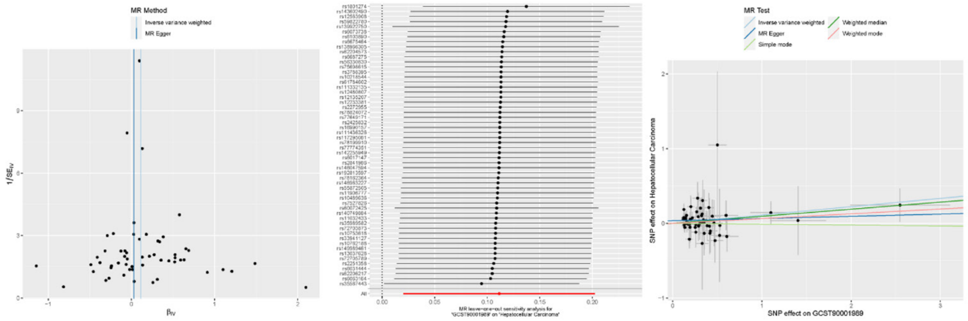

GCST90002030

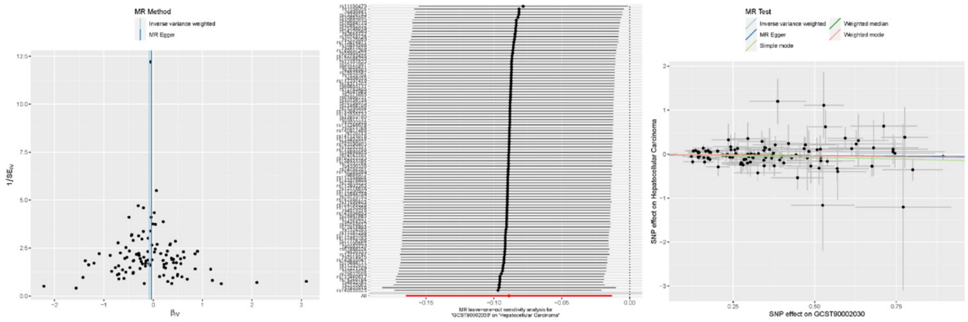

Supplementary Figure 1. (*Continued*).

GCST90002043

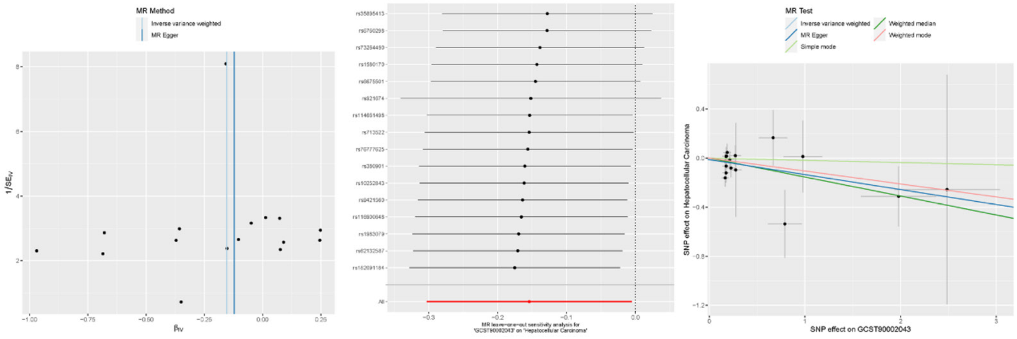

GCST90002085

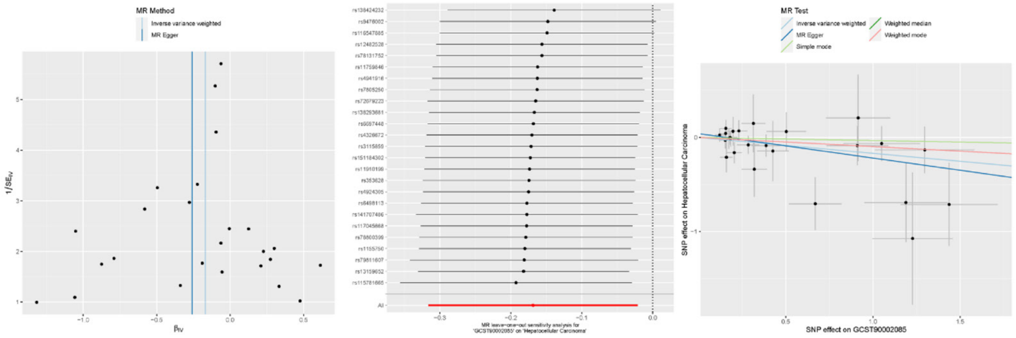

GCST90002104

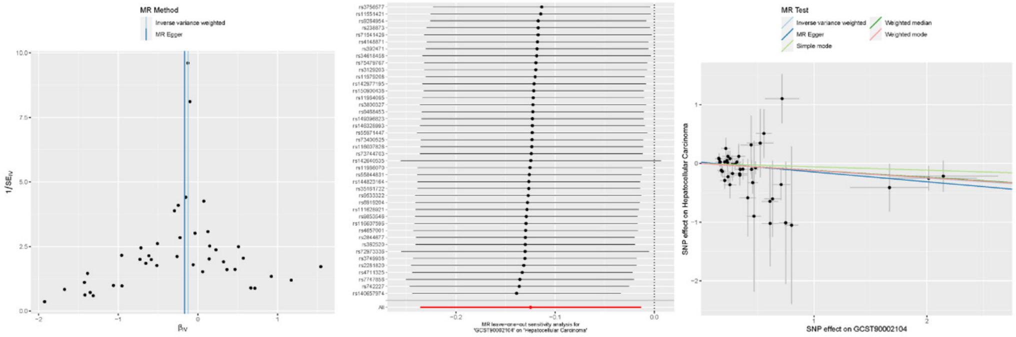

GCST90002109

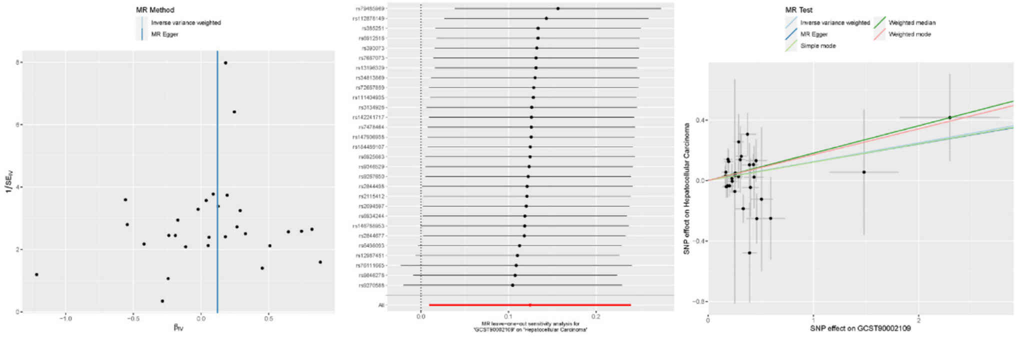

Supplementary Figure 1. (*Continued*).

GCST90001422

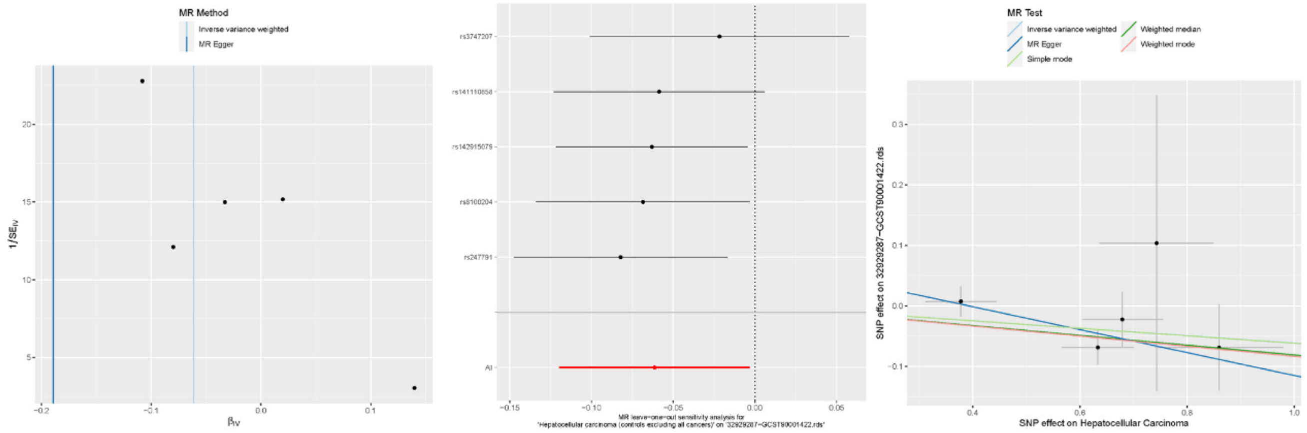

GCST90001444

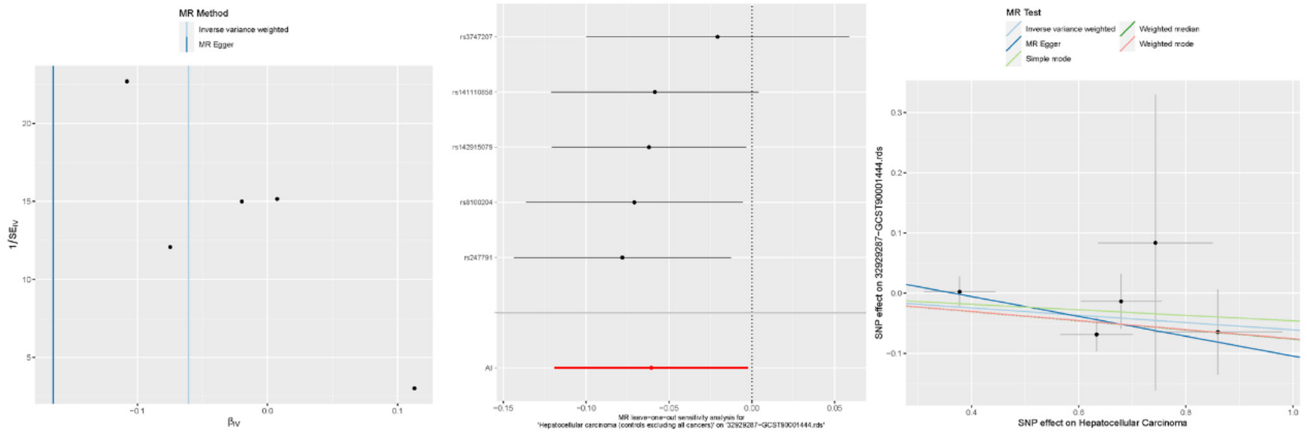

GCST90001516

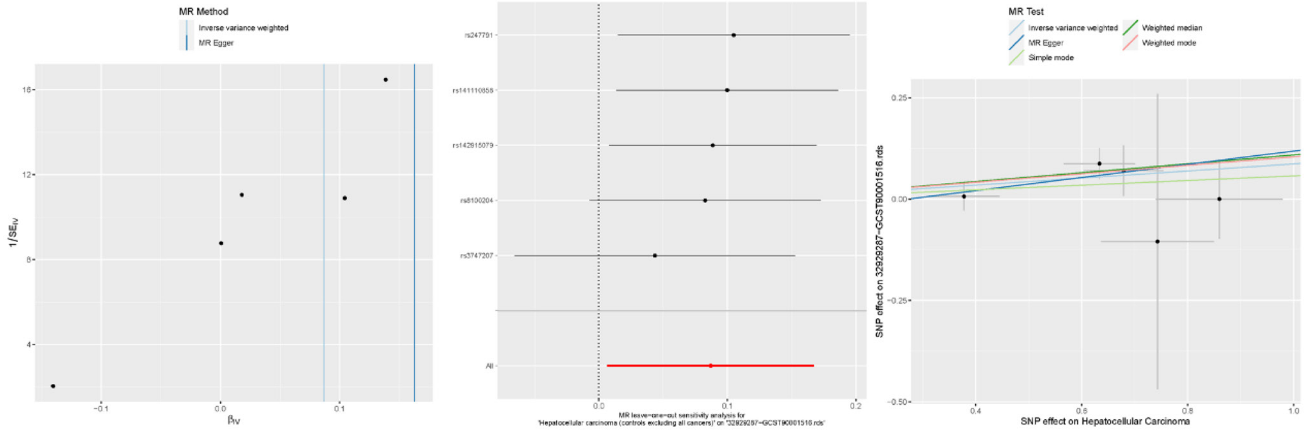

Supplementary Figure 2. Sensitivity Analyses for reverse MR Results of HCC and Immune Traits. This figure presents the sensitivity analyses conducted to validate the MR results for the causal relationships between HCC and the 11 identified immune traits. Scatter plots, leave-one-out forest plots, and funnel plots were used to assess the robustness of the MR estimates and identify potential biases.

GCST90001691

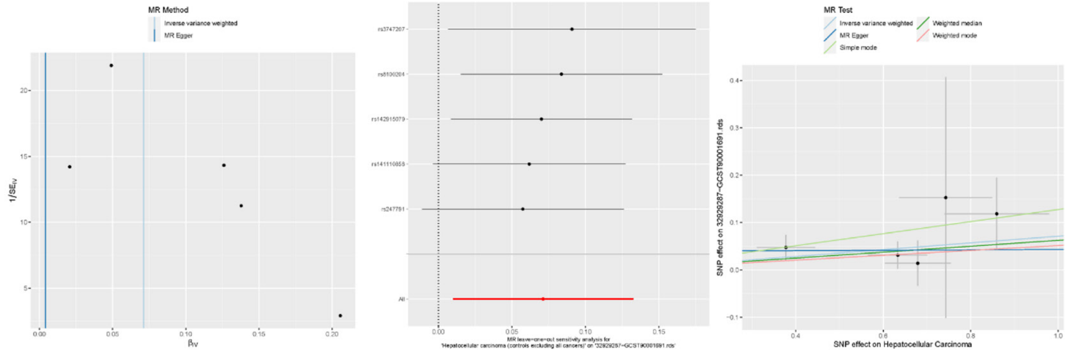

GCST90001888

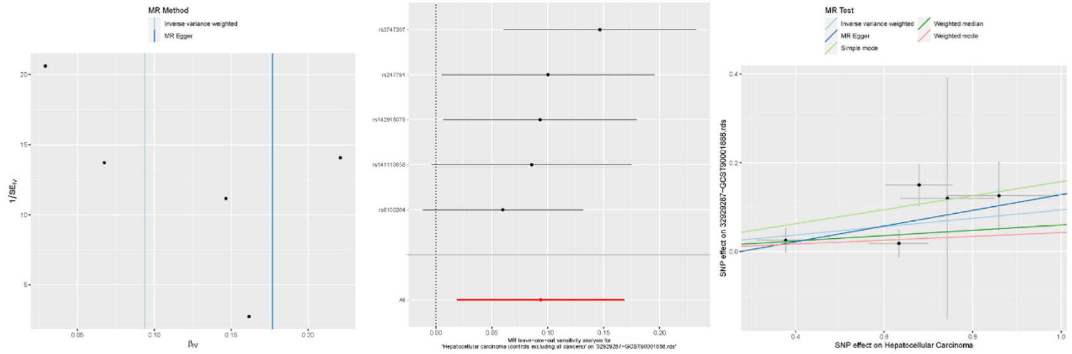

GCST9002013

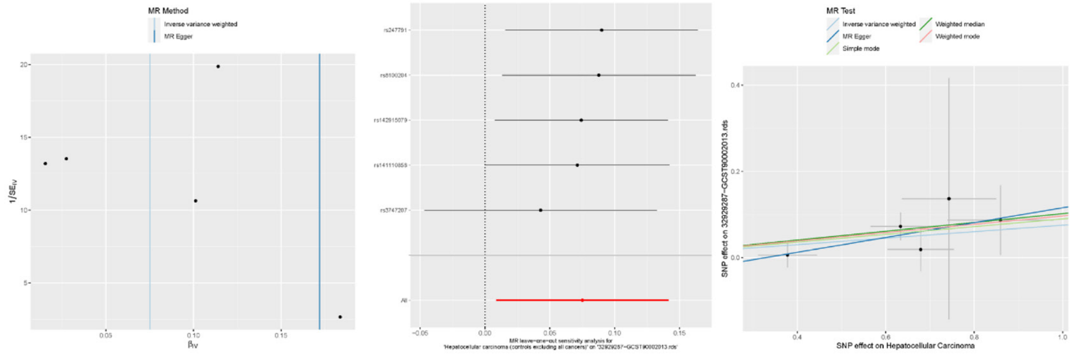

GCST90002014

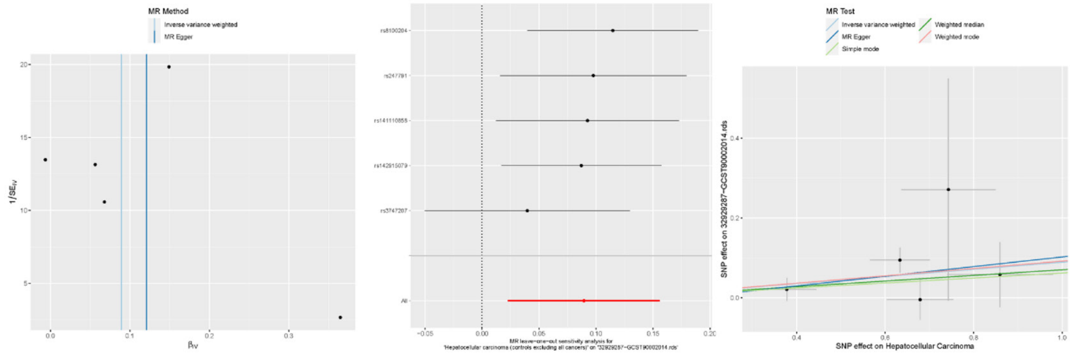

Supplementary Figure 2. (*Continued*).

GCST90002069

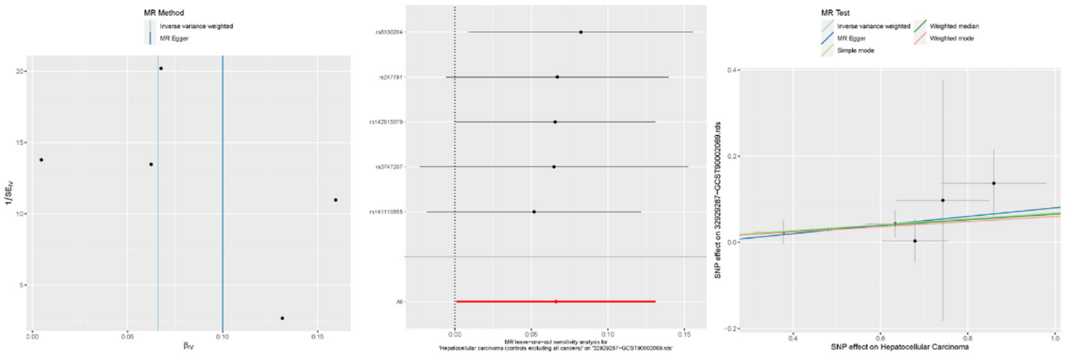

GCST9002101

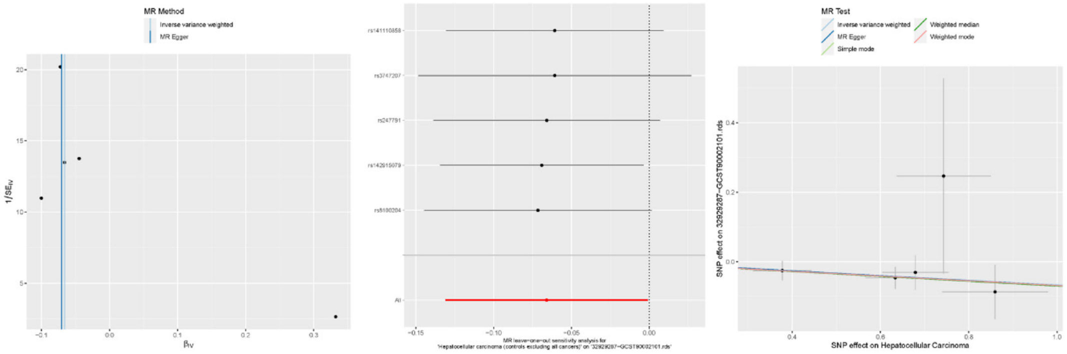

GCST90002113

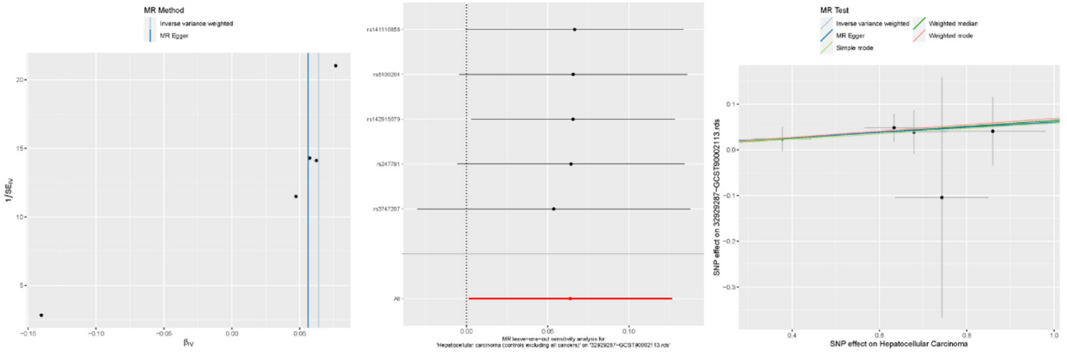

GCST90002115

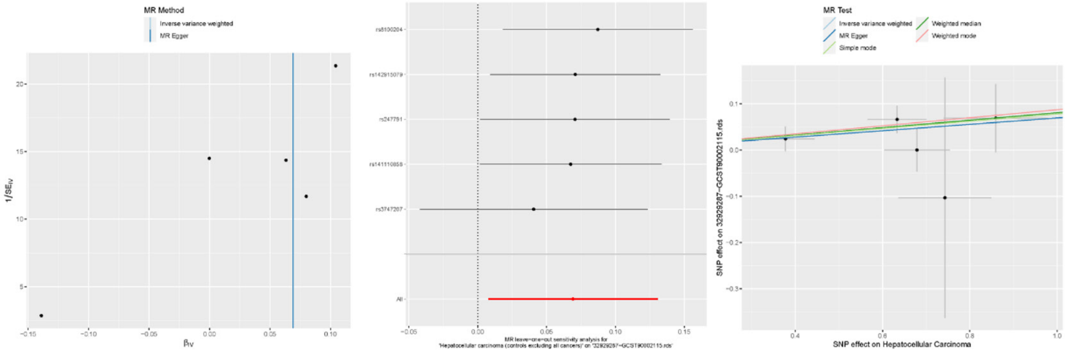

Supplementary Figure 2. (*Continued*).
